# Supplementary material for: Resolving the full spectrum of human genome variation using Linked-Reads
Source: Genome Res. 2019 Apr;29(4):635–45. doi: 10.1101/gr.234443.118 (PMC6442396; doi:10.1101/gr.234443.118)
Supplement: Supplemental Material [file supp_gr.234443.118_Supplemental_longranger-2.2.zip › longranger-2.2.tar.bz2!/lr2.2-public/lib/stan/cmdstan/stan/doc/api/html/rev_2functions_2fma_8hpp_source.html]

Stan: src/stan/agrad/rev/functions/fma.hpp Source File


|  |  |
| --- | --- |
|  | Stan  2.5.0  probability, sampling & optimization |


- Main Page
- Related Pages
- Namespaces
- Classes
- Files

- File List
- File Members

All Classes Namespaces Files Functions Variables Typedefs Enumerations Enumerator Friends Macros Pages

- src
- stan
- agrad
- rev
- functions

fma.hpp

Go to the documentation of this file.

1 #ifndef STAN\_\_AGRAD\_\_REV\_\_FUNCTIONS\_\_FMA\_HPP

2 #define STAN\_\_AGRAD\_\_REV\_\_FUNCTIONS\_\_FMA\_HPP

3

4 #include <cmath>

5 #include <valarray>

6 #include <stan/agrad/rev/var.hpp>

7 #include <stan/agrad/rev/internal/ddv\_vari.hpp>

8 #include <stan/agrad/rev/internal/vdd\_vari.hpp>

9 #include <stan/agrad/rev/internal/vvv\_vari.hpp>

10 #include <stan/agrad/rev/internal/vvd\_vari.hpp>

11 #include <stan/agrad/rev/internal/vdv\_vari.hpp>

12 #include <stan/math/constants.hpp>

13 #include <boost/math/special\_functions/fpclassify.hpp>

14 #include <stan/meta/likely.hpp>

15

16 namespace stan {

17  namespace agrad {

18

19  namespace {

20  class fma\_vvv\_vari : public op\_vvv\_vari {

21  public:

22  fma\_vvv\_vari(vari\* avi, vari\* bvi, vari\* cvi) :

23  op\_vvv\_vari(::fma(avi->val\_, bvi->val\_, cvi->val\_),

24  avi,bvi,cvi) {

25  }

26  void chain() {

27  if (unlikely(boost::math::isnan(avi\_->val\_)

28  || boost::math::isnan(bvi\_->val\_)

29  || boost::math::isnan(cvi\_->val\_))) {

30  avi\_->adj\_ = std::numeric\_limits<double>::quiet\_NaN();

31  bvi\_->adj\_ = std::numeric\_limits<double>::quiet\_NaN();

32  cvi\_->adj\_ = std::numeric\_limits<double>::quiet\_NaN();

33  } else {

34  avi\_->adj\_ += adj\_ \* bvi\_->val\_;

35  bvi\_->adj\_ += adj\_ \* avi\_->val\_;

36  cvi\_->adj\_ += adj\_;

37  }

38  }

39  };

40

41  class fma\_vvd\_vari : public op\_vvd\_vari {

42  public:

43  fma\_vvd\_vari(vari\* avi, vari\* bvi, double c) :

44  op\_vvd\_vari(::fma(avi->val\_, bvi->val\_, c),

45  avi,bvi,c) {

46  }

47  void chain() {

48  if (unlikely(boost::math::isnan(avi\_->val\_)

49  || boost::math::isnan(bvi\_->val\_)

50  || boost::math::isnan(cd\_))) {

51  avi\_->adj\_ = std::numeric\_limits<double>::quiet\_NaN();

52  bvi\_->adj\_ = std::numeric\_limits<double>::quiet\_NaN();

53  } else {

54  avi\_->adj\_ += adj\_ \* bvi\_->val\_;

55  bvi\_->adj\_ += adj\_ \* avi\_->val\_;

56  }

57  }

58  };

59

60  class fma\_vdv\_vari : public op\_vdv\_vari {

61  public:

62  fma\_vdv\_vari(vari\* avi, double b, vari\* cvi) :

63  op\_vdv\_vari(::fma(avi->val\_ , b, cvi->val\_),

64  avi,b,cvi) {

65  }

66  void chain() {

67  if (unlikely(boost::math::isnan(avi\_->val\_)

68  || boost::math::isnan(cvi\_->val\_)

69  || boost::math::isnan(bd\_))) {

70  avi\_->adj\_ = std::numeric\_limits<double>::quiet\_NaN();

71  cvi\_->adj\_ = std::numeric\_limits<double>::quiet\_NaN();

72  } else {

73  avi\_->adj\_ += adj\_ \* bd\_;

74  cvi\_->adj\_ += adj\_;

75  }

76  }

77  };

78

79  class fma\_vdd\_vari : public op\_vdd\_vari {

80  public:

81  fma\_vdd\_vari(vari\* avi, double b, double c) :

82  op\_vdd\_vari(::fma(avi->val\_ , b, c),

83  avi,b,c) {

84  }

85  void chain() {

86  if (unlikely(boost::math::isnan(avi\_->val\_)

87  || boost::math::isnan(bd\_)

88  || boost::math::isnan(cd\_)))

89  avi\_->adj\_ = std::numeric\_limits<double>::quiet\_NaN();

90  else

91  avi\_->adj\_ += adj\_ \* bd\_;

92  }

93  };

94

95  class fma\_ddv\_vari : public op\_ddv\_vari {

96  public:

97  fma\_ddv\_vari(double a, double b, vari\* cvi) :

98  op\_ddv\_vari(::fma(a, b, cvi->val\_),

99  a,b,cvi) {

100  }

101  void chain() {

102

103  if (unlikely(boost::math::isnan(cvi\_->val\_)

104  || boost::math::isnan(ad\_)

105  || boost::math::isnan(bd\_)))

106  cvi\_->adj\_ = std::numeric\_limits<double>::quiet\_NaN();

107  else

108  cvi\_->adj\_ += adj\_;

109  }

110  };

111  }

112

134  inline var fma(const stan::agrad::var& a,

135  const stan::agrad::var& b,

136  const stan::agrad::var& c) {

137  return var(new fma\_vvv\_vari(a.vi\_,b.vi\_,c.vi\_));

138  }

139

159  inline var fma(const stan::agrad::var& a,

160  const stan::agrad::var& b,

161  const double& c) {

162  return var(new fma\_vvd\_vari(a.vi\_,b.vi\_,c));

163  }

164

184  inline var fma(const stan::agrad::var& a,

185  const double& b,

186  const stan::agrad::var& c) {

187  return var(new fma\_vdv\_vari(a.vi\_,b,c.vi\_));

188  }

189

207  inline var fma(const stan::agrad::var& a,

208  const double& b,

209  const double& c) {

210  return var(new fma\_vdd\_vari(a.vi\_,b,c));

211  }

212

230  inline var fma(const double& a,

231  const stan::agrad::var& b,

232  const double& c) {

233  return var(new fma\_vdd\_vari(b.vi\_,a,c));

234  }

235

253  inline var fma(const double& a,

254  const double& b,

255  const stan::agrad::var& c) {

256  return var(new fma\_ddv\_vari(a,b,c.vi\_));

257  }

258

278  inline var fma(const double& a,

279  const stan::agrad::var& b,

280  const stan::agrad::var& c) {

281  return var(new fma\_vdv\_vari(b.vi\_,a,c.vi\_)); // a-b symmetry

282  }

283

284  }

285 }

286 #endif

stan::agrad::fma

fvar< typename stan::return\_type< T1, T2, T3 >::type > fma(const fvar< T1 > &x1, const fvar< T2 > &x2, const fvar< T3 > &x3)

The fused multiply-add operation (C99).

**Definition:** fma.hpp:62

boost::math::isnan

bool isnan(const stan::agrad::var &v)

Checks if the given number is NaN.

**Definition:** boost\_fpclassify.hpp:69

vvd\_vari.hpp

vdd\_vari.hpp

stan::agrad::var::vi\_

vari \* vi\_

Pointer to the implementation of this variable.

**Definition:** var.hpp:40

unlikely

#define unlikely(x)

**Definition:** likely.hpp:9

vvv\_vari.hpp

var.hpp

likely.hpp

stan::agrad::var

Independent (input) and dependent (output) variables for gradients.

**Definition:** var.hpp:27

vdv\_vari.hpp

constants.hpp

ddv\_vari.hpp


---

[ Stan Home Page ]

*© 2011–2014,
Stan Development Team.*
